# Pes Anserinus Structural Framework and Constituting Tendons Are Grossly Aberrant in Nigerian Population

**DOI:** 10.1155/2015/483186

**Published:** 2015-07-09

**Authors:** J. O. Ashaolu, T. S. Osinuga, V. O. Ukwenya, E. O. Makinde, A. J. Adekanmbi

**Affiliations:** ^1^Department of Anatomy, Faculty of Basic Medical Sciences, College of Health Sciences, Bowen University, Iwo 284, Osun State, Nigeria; ^2^Department of Anatomy, Faculty of Basic Medical Sciences, College of Health Sciences, University of Ilorin, Ilorin 1515, Kwara State, Nigeria; ^3^Department of Anatomy, Faculty of Basic Medical Sciences, College of Medicine, Ekiti State University, Ado-Ekiti 5363, Ekiti State, Nigeria

## Abstract

We evaluated the morphological framework of the pes anserinus in both knees of ten Nigerian cadavers and we observed high degree of variability in its morphology and location. The pes anserinus inserted specifically on the superior half of the media border of the tibia, as far inferiorly as 124.44 mm to the tibial tuberosity (prolonged insertion). The insertion was also joined to the part of tibia close to the tibia tuberosity (90%) and to the fascia cruris (10%). The initial insertion point of the pes anserinus was always found at the level of the tibia tuberosity. We found out that accessory bands of sartorius, gracilis, or semitendinosus were part of the pes anserinus in 95% of all occasions studied whereas the combined occurrence of monotendinosus sartorius, gracilis, and semitendinosus tendons was found in only 5% of all occasions. The pes anserinus did not conform to the layered pattern and the tendons of sartorius, gracilis, or semitendinosus were short. The inferior prolongation of the pes anserinus connotes extended surface area of attachment to support the mechanical pull from the hamstring muscles. This information will be useful in precise location and grafting of the pes anserinus.

## 1. Introduction

Pes anserinus is a confluence of tendinous structures on the medial side of knee and leg [1, 2]. Its name means “goose foot” because of the radiating pattern of the tendons that constitute it [1, 2]. The pes anserinus is commonly said to be constituted by the tendons of three anatomical structures, namely, sartorius, gracilis, and semitendinosus muscles [2]. However, some authors have indicated that other musculotendinous structures may participate in its formation [1, 2]. Surgical excision of pes anserinus is used in a wide range of reconstructive procedures, including augmentation in Achilles tendon repairs, patellar tendon repairs after chronic ruptures, patellar subluxation procedures, replacement of damaged anterior cruciate ligament (ACL), and surgical grafting for medial knee reinforcement (pes anserinus transplantation) [1, 2]. Other times, pes anserine bursitis occurs and correct surgical marking of the pes anserinus and its bursa is important before treatment could be performed. Morphometric information of the constituting tendons can also aid surgical decisions that favor successful grafting outcome. Kartus et al. [3] reported that surgical incisions placed too close to the tibial tuberosity or the apex of patella may injure the infrapatellar branch of the saphenous nerve. Varying insertion pattern, location, and constituting tendon measurements have been reported in different populations while until now dearth of information is found regarding African subjects.

This current study presents new methods for the classification of the pes anserinus and aims to determine the constituting structures, relative locations, and morphometric values of the tendons of pes anserinus in the African population using Nigeria as a case study.

## 2. Materials and Methods

Twenty (20) adult knees (10 left and 10 right) were sourced from the Anatomy Department of the College of Health Sciences, Bowen University, Iwo, Osun State, Nigeria. The experimental procedure received ethical approval from the Board of Research Ethics, College of Health Science, Bowen University. The knees that were used were previously undissected; they were fixated in 10% formaldehyde, glycerol, liquid phenol, acetic acid, and ethyl alcohol. Median incisions were made at the anterior and posterior part of the thigh and leg. The incision along the posterior aspect extended from the mid gluteal fold to the calcaneus whereas that on the anterior aspect of the leg extended from the inguinal ligament to the point between the medial and lateral malleoli. Transverse incisions were made around the circumference of the knees at the level of the joint line. Then skin and superficial fascia were carefully reflected, followed by reflection of the fascia lata and fascia cruris. The assessment of the muscles and pes anserinus structures commenced afterwards.

### 2.1. Descriptive Evaluation of Pes Anserinus

#### 2.1.1. Constituting Tendons

For each pes anserinus studied, the constituting tendons or ligaments were recorded after examining the superior, internal, and posterior aspects of the pes anserinus for possible attachments.

#### 2.1.2. Tendon Convergence Pattern

The modes of termination of the tendons that constituted the pes anserinus were classified as

- early convergence: when the tendons unite to form the pes anserinus superior to medial tibial condyle,
- late convergence: when the tendons unite inferior to the medial tibial condyle,
- nonconvergence: when they have side-by-side termination.

#### 2.1.3. Mode of Insertion

The point of termination of pes anserinus was also determined and specific points observed were superior anteromedial tibia, medial border of tibia, and fascia cruris.

### 2.2. Quantitative Evaluation of Pes Anserinus

#### 2.2.1. Horizontal and Vertical Dimensions of Pes Anserinus Relative to Tibial Tuberosity

Linear measurement was conducted using Mitutoyo Micrometer (Japan, B.7502) and Vernier Calliper. Minimum and maximum distances of the pes anserinus were determined relative to the inferior border of tibial tuberosity both vertically and horizontally. The mediolateral distance (breadth) of the pes anserinus was measured at the level of the inferior border of tibial tuberosity while the proximodistal (length) distance was measured from the most superior point of tibial attachment to the most inferior point of tibial attachment.

#### 2.2.2. Tendon Lengths and Thicknesses of Constituting Tendons of Pes Anserinus

The length and thickness of the sartorius, semitendinosus, and gracilis tendons of pes anserinus were measured. The individual tendon length was measured from the distal end of muscle mass to the point where it conjoined to other tendons of the pes anserinus or its distal attachment (if directly attached to bone).

### 2.3. Statistical Analysis

Statistical comparisons were performed using Paired Sample *t*-test to compare values between right and left legs and between males and females. SPSS statistical software (version 20) was used. *P* value < 0.05 was considered statistically significant.

## 3. Results

### 3.1. Descriptive Analysis of Pes Anserinus

#### 3.1.1. Constituting Tendons

**Figure 1:**
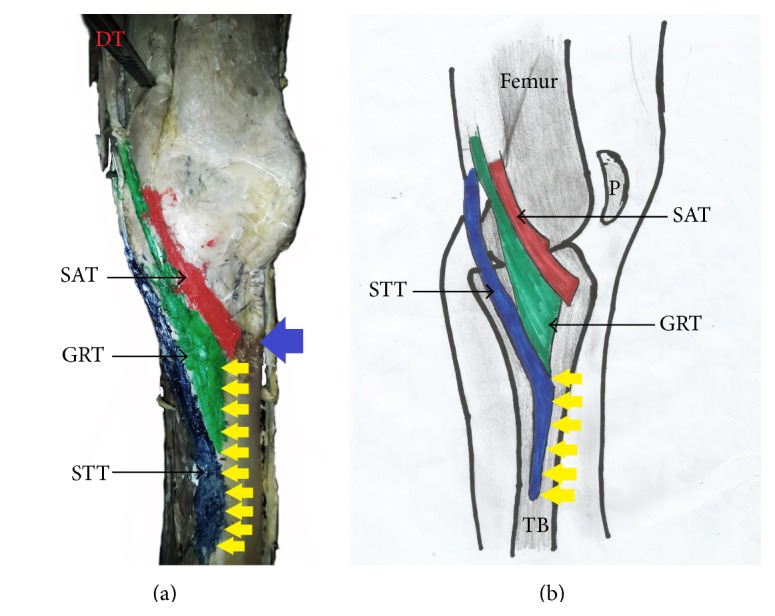
(a) Photograph and (b) schematic illustration showing painted type I pes anserinus with side-by-side (nonconvergent) termination of sartorius, gracilis, and semitendinosus tendons. DT: dissection tool; SAT: sartorius tendon; GRT: gracilis tendon; STT: semitendinosus tendon; blue arrow indicates tibial tuberosity; yellow arrow indicates inferior attachment of pes anserinus; P: patella; TB: tibia.

**Figure 2:**
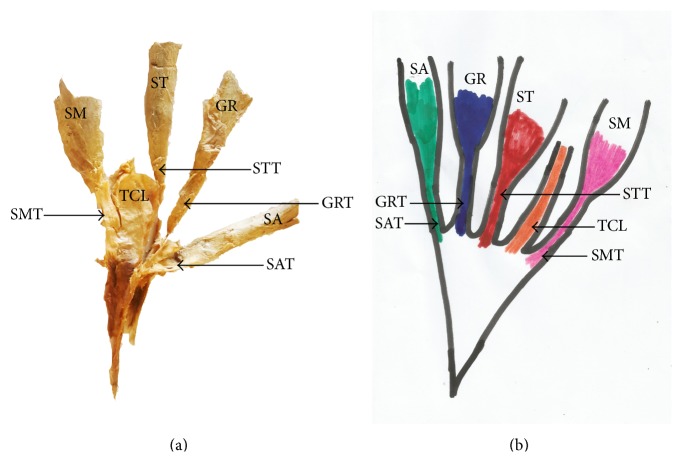
Photograph showing detached cadaveric type II pes anserinus with accessory participation from semimembranosus tendon and tibial collateral ligament. SA: sartorius muscle; SM: semimembranosus muscle; GR: gracilis muscle; SAT: sartorius tendon; GRT: gracilis tendon; STT: semitendinosus tendon; SMT: semimembranosus tendon; TCL: tibial collateral ligament.

**Figure 3:**
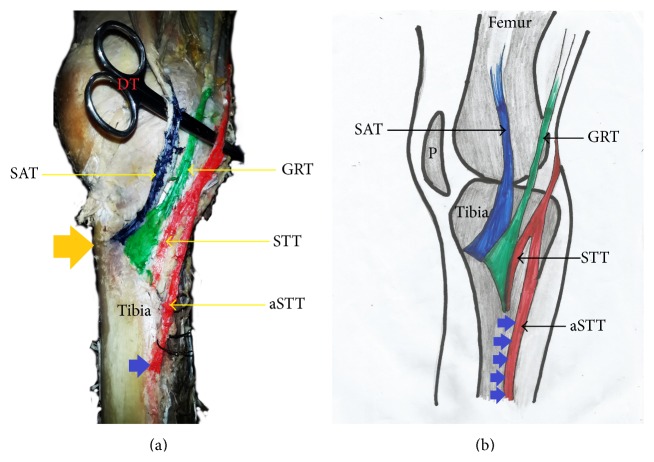
(a) Photograph and (b) schematic illustration showing painted type III pes anserinus formed by sartorius, gracilis, and semitendinosus tendons and an accessory semitendinosus tendon gracilis and semitendinosus. Each constituting tendon attached as separate entity on the tibia. Hence, this pes anserinus type is said to exhibit the nonconvergence (side-by-side termination) pattern based on the union of constituting tendons classification of pes anserinus. DT: dissection tool; SA: sartorius muscle; SAT: sartorius tendon; GRT: gracilis tendon; STT: semitendinosus tendon; aSTT: accessory semitendinosus tendon; orange arrow indicates level of tibial tuberosity; P: patella; blue arrow indicates the inferior attachment of pes anserinus.

**Figure 4:**
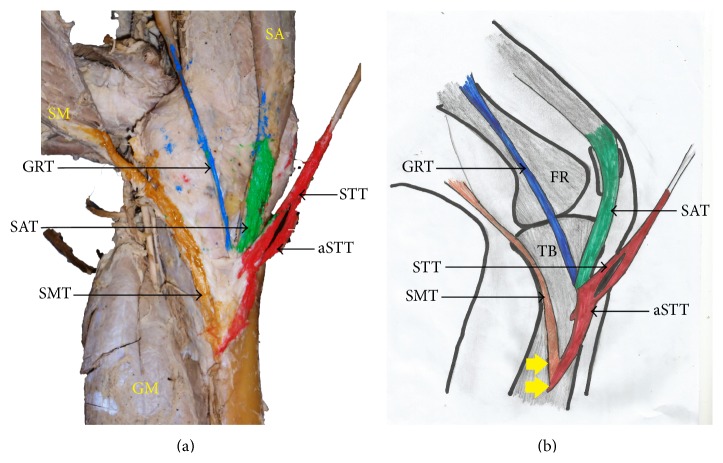
(a) Photograph and (b) schematic illustration showing the internal aspect of a painted type IV pes anserinus formed by sartorius, gracilis, and semitendinosus tendons with additional participation from an accessory semitendinosus tendon and semimembranosus tendon. Note: the attachment of this pes anserinus extends beyond the superior anteromedial tibia. SA: sartorius muscle; SM: semimembranosus muscle; GM: gastrocnemius muscle; SAT: sartorius tendon; GRT: gracilis tendon; STT: semitendinosus tendon; aSTT: accessory semitendinosus tendon; SMT: semimembranosus tendon; yellow arrow indicates inferior attachment of pes anserinus.

**Figure 5:**
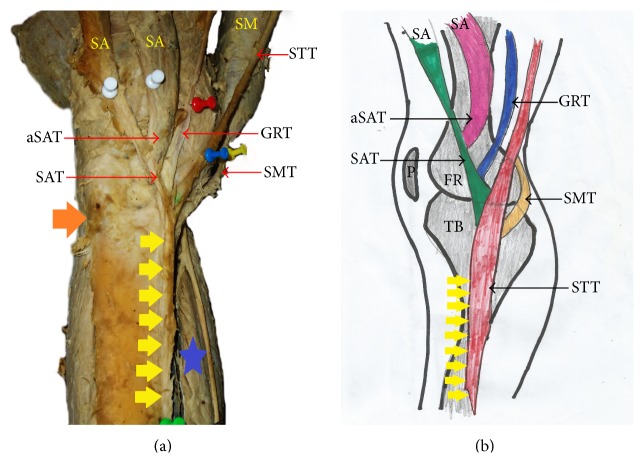
(a) Photograph and (b) schematic illustration showing type V pes anserinus with accessory participation from a second sartorius muscle and semitendinosus tendon. SA: sartorius muscle; SM: semimembranosus muscle; SAT: sartorius tendon; aSAT: accessory sartorius tendon; GRT: gracilis tendon; STT: semitendinosus tendon; SMT: semimembranosus tendon; orange arrow indicates tibial tuberosity; yellow arrow indicates inferior attachment of pes anserinus; blue star indicates gastrocnemius muscle; FR: femur.

**Figure 6:**
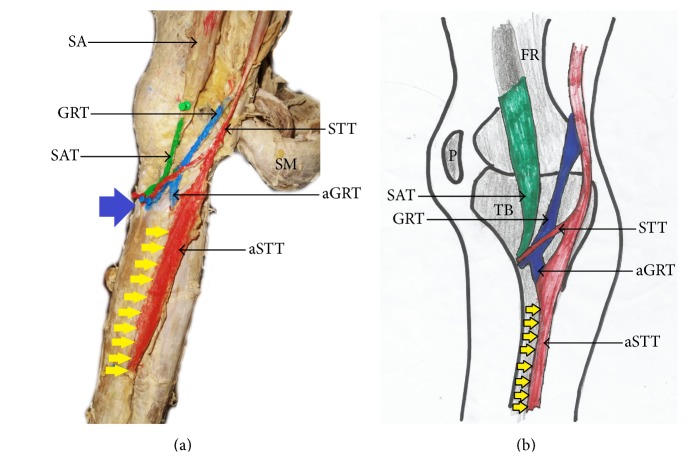
(a) Photograph and (b) schematic illustration of type VI pes anserinus showing accessory tendons of gracilis and semitendinosus participation. SA: sartorius muscle; SM: semimembranosus muscle; FR: femur; TB: tibia; SAT: sartorius tendon; GRT: gracilis tendon; aGRT: accessory gracilis tendon; STT: semitendinosus tendon; aSTT: accessory semitendinosus tendon; yellow arrow indicates inferior attachment of pes anserinus.

The pes anserinus was constituted by monotendinosus sartorius, gracilis, and semitendinosus only (type I) in 5% of the studied population (Figure 1 and Table 1). In the remaining 95% of the subjects, there was participation from other structures (Figures 2–6 and Table 1). Tibial collateral ligament contributed to pes anserinus formation (type II) in 5% of all occasions (Figure 1 and Table 1) The most common pattern (65%) observed was the combined constitution by sartorius, gracilis, semitendinosus, and accessory semitendinosus tendon (type III) (Figure 3 and Table 1). Accessory semitendinosus band (aST) was seen in 90% of all occasions (types II, III, IV, and VI) (Figures 2–6). Semimembranosus tendon participated (types IV and V) in 25% of pes anserinus formation (Figures 4 and 5). The combined existence of accessory sartorius band (aSAT) and accessory gracilis band (aGAT) (type VI) was recorded in 5% of the studied population (Figure 6 and Table 1).

#### 3.1.2. Tendon Convergence Pattern

**Figure 7:**
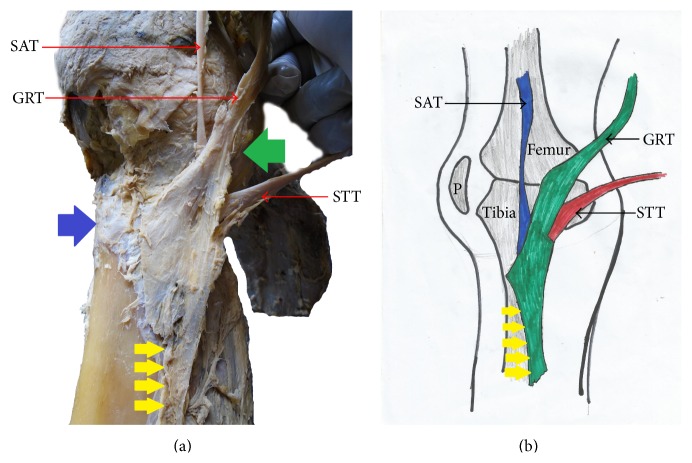
(a) Photograph and (b) schematic illustration showing pes anserinus exhibiting the late convergence pattern. This late convergence pattern of pes anserinus shows the constituting tendons of the pes anserinus complex converging below the medial tibial condyle. This pes anserinus also has attachment to the medial border of tibia. SAT: sartorius tendon; GRT: gracilis tendon; STT: semitendinosus tendon; green arrow indicates the level of the medial femoral condyle; blue arrow indicates the tibial tuberosity; P: patella; yellow arrow indicates inferior attachment of pes anserinus.

**Figure 8:**
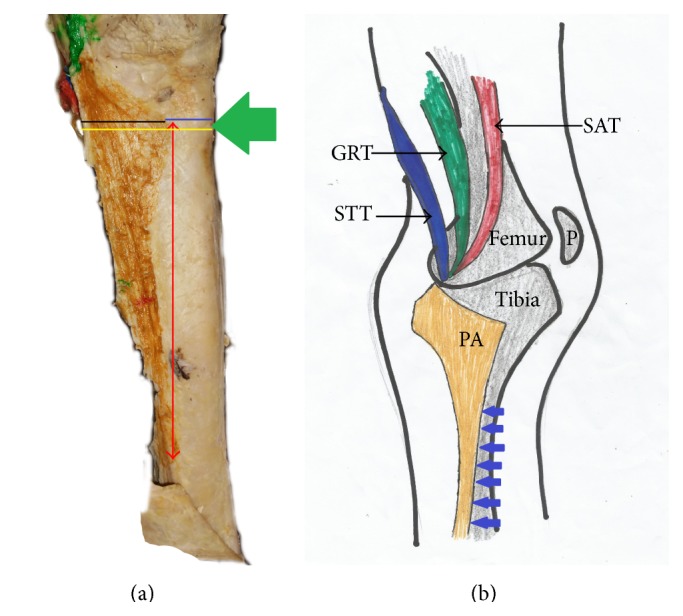
(a) Photograph and (b) schematic illustration showing painted pes anserinus exhibiting the early convergence pattern. Green arrow indicates the level of the tibial tuberosity; red double headed arrow indicates the lowest insertion point relative to tibial tuberosity; black line indicates the mediolateral distance; yellow line indicates the maximum horizontal distance; blue line indicates the minimum horizontal distance; SAT: sartorius tendon; GRT: gracilis tendon; STT: semitendinosus tendon; PA: pes anserinus; P: patella; blue arrow indicates inferior attachment of pes anserinus.

The tendons which constitute the pes anserinus converged inferior to medial tibial condyle (late convergence) in 85% of the occasions (Figure 7 and Table 1), but cases of early convergence were observed only in 5% of occasions (Figure 8 and Table 1). Nonconvergent pes anserinus (side-by-side termination) was observed in 10% of all occasions (Figure 1 and Table 1). When the pes anserinus tendons converge, they become one entity and are inseparable. Thus, the layered formation was not observed.

#### 3.1.3. Mode of Insertion

The attachment of the pes anserinus was found to be on both the superior anteromedial tibia and medial border of tibia in 90% of all occasions studied (Figures 1–8 and Table 1). In the remaining 10%, it attached to the trio (superior anteromedial tibia + medial border of tibia + fascia cruris) (Table 1). Pes anserinus did not attach to the superior anteromedial tibia only.

### 3.2. Quantitative Analysis of Pes Anserinus

#### 3.2.1. Horizontal and Vertical Dimensions of Pes Anserinus Relative to Tibial Tuberosity

The mean minimum and maximum horizontal distance of pes anserinus to tibial tuberosity were 24.96 mm and 64.96 mm, respectively (Table 2 and Figure 7). The mean minimum and maximum horizontal distance of pes anserinus were not significantly different between right side (23.29 mm and 63.37 mm, resp.) and the left side of the body (26.63 mm and 66.68 mm, resp.) (Table 2).

Relating to gender, it was observed that the mean minimum horizontal distance was not significantly different between males (23.60 mm) and females (28.64 mm). The mean maximum horizontal distance was not significantly different between males (65.09 mm) and females (64.88 mm) and between right and left side of the body (Table 2 and Figure 7).

The mediolateral distance at the level of tibial tuberosity was 40.36 mm while the proximodistal distance of the pes anserinus at the point of attachment to the tibia is 134.02 mm. Both the mean proximodistal and mediolateral distances were not significantly different between males and females (proximodistal distance: males = 139.50 mm, females = 121.21 mm; mediolateral distance: males = 41.57 mm, females = 37.54 mm). It was also observed that both distances were not significantly different between right and left legs (proximodistal distance: right = 128.11 mm, left = 139.92 mm; mediolateral distance: right = 40.23 mm, left = 40.50 mm) (Table 2).

The initial insertion point of the pes anserinus was always found at the level of the tibia tuberosity. The mean lowest insertion point of pes anserinus relative to tibial tuberosity on average in the studied specimen was 124.44 mm (Table 2). The mean lowest insertion point of the pes anserinus relative to tibial tuberosity is not significantly different between males (121.00 mm) and females (130.97 mm) (Table 2). However, on the left side it was significantly higher (127.68 mm) than on the right side (120.30 mm) (Table 2).

#### 3.2.2. Tendon Length and Thicknesses of Constituting Tendons of Pes Anserinus

We also found considerable differences in the length and thickness of the sartorius, gracilis, and semitendinosus tendons (Table 3). The mean length of the sartorius tendon was 52.66 mm with a thickness of 1.49 mm. The mean length of the gracilis tendon was 116.07 mm with a thickness of 1.92 mm (Table 3). The mean length of the semitendinosus tendon was 116.80 mm with a thickness of 2.66 mm (Table 3).

The length and thickness of gracilis, sartorius, and semitendinosus tendon length were not significantly different between males and females (Table 3).

## 4. Discussion

In the current study, we defined three means of classification of pes anserinus because of its high degree of variability. It was classified based on the point of attachment in the leg, mode of the convergence of the constituting tendons, and the individual tendon that constituted the pes anserinus complex.

The main findings of the study were as follows:

- The pes anserinus inserted approximately on the superior medial border of the tibia, as far inferiorly as 124.44 mm to the tibial tuberosity. The attachment joined close to the tibial tuberosity (90%) and to the fascia cruris (10%).
- The accessory bands of sartorius, gracilis, or semitendinosus were part of the pes anserinus in 95% of all specimens studied whereas the combined occurrence of monotendinosus sartorius, gracilis, and semitendinosus tendons was found only in 5% of all occasions. Unusual participations were also seen from semimembranosus tendon and tibial collateral ligament.
- The pes anserinus did not conform to the layered pattern.
- The initial insertion point of the pes anserinus was always found at the level of the tibia tuberosity.
- The tendons of sartorius, gracilis, and semitendinosus were shorter than those reported in other populations.

Our current study has demonstrated that the pes anserinus did not attach only on superior anteromedial tibia but commonly extended downward to attach on the superior medial border of tibia. Most authors have consistently described the insertion of the pes anserinus as being on the superior anteromedial tibia only [4–6]. This inferior prolongation of the pes anserinus is also evident in the high value of the lowest insertion point (124.44 mm) of the pes anserinus relative to the tibial tuberosity. In some other occasions (10%), it additionally joins the fascia cruris and could serve as a tensor of the fascia cruris. The inferior prolongation of the pes anserinus connotes extended surface area of attachment to support the mechanical pull from the hamstring muscles.

Another observation made was that the length of the tendons of pes anserinus (sartorius: 50.66 mm, gracilis: 116.07 mm, and semitendinosus: 116.80 mm) was shorter when compared to other studied populations. In the Austrian population, the length of the semitendinosus tendon was 263 mm whereas that of gracilis tendon was 232.5 mm [7]. This could be as a result of the bulky and longer muscular mass of the hamstrings muscles seen in our own population. Again, thicker tendons were seen being 1.49 mm for sartorius, 1.92 mm for gracilis, and 2.66 mm for semitendinosus which may provide mechanical restrain and compensate for the shortness of the tendons.

To be able to minimize skin incision, reduce donor site morbidity, and lessen extensor mechanism dysfunction in pes anserinus graft, it is essential that the surgeon have an understanding of the surface markings of the pes anserinus. In the current study, we reported that the minimum and maximum horizontal distances to the tibial tuberosity were 24.96 mm (10.06 mm–39.40 mm) and 65.03 mm (48.99–88.92 mm), respectively. We also reported the lowest insertion points of the pes anserinus to tibial tuberosity as 124.44 mm (24.71–222.77 mm) whereas the initial insertion of pes anserinus was consistently at the level of tibial tuberosity. Pagnani et al. [8] reported the minimum vertical distance (initial insertion point) of the pes anserinus to the tibial tuberosity to be 19 mm (10–25 mm) and the minimum horizontal distance to the tibial tuberosity to be 22.5 mm (13–30 mm). Kijkunasathian et al. [9] in their study on Thai population reported the minimum vertical distance as 5.0 mm (0–20 mm), whereas the minimum horizontal distance was 25 mm (10–40 mm). Marking the location of the pes anserinus is the first surgical step in conducting successful pes anserinus graft and can prevent damage to the infrapatellar branch of the saphenous nerve.

The mode of convergence of the constituting tendons of pes anserinus was such that, in 85% of the subjects, there was convergence inferior to the medial tibial condyle. Convergences were also seen in some cases (5%) above the medial tibial condyle. In these specimens, it will be difficult to target any individual tendon for harvesting. In 10% of the specimens, there was side-by-side termination (nonconvergence) on the tibia. With this pattern, harvesting will be the easiest.

Another factor that can determine the success of pes anserinus grafting is the presence of supernumerary tendons. This can occur as accessory bands of semitendinosus, gracilis, sartorius, semimembranosus, or contributions from tibial collateral ligament. We found accessory bands in 95% of this study, and this trend is consistent with the formation reported by previous studies [8, 10, 11]. The pattern of the accessory bands that we found was such that they diverged from the main tendons close to their insertion on the tibia projecting further inferiorly.

Other authors have reported that accessory bands can also pass between the semitendinosus and gracilis tendons or between hamstrings or pes anserinus tendons or to gastrocnemius, popliteal, pretibial, and superficial fascia [10]. Recognizing these accessory bands and dividing them are essential. If these bands are not recognized and divided, they can divert the tendon striper into the main tendon leading to premature tendon amputation and short graft [5]. Care must be taken to avoid damaging the tibial collateral ligament and semimembranosus tendon because they also participate in the pes anserinus formation as seen in this work. Most of the earlier studies have merely reported a close proximity of tibial collateral ligament to pes anserinus but this current study showed that the tibial collateral ligament also participated. The damage to the tibial collateral ligament during tendon grafting can lead to instability of the medial knee region.

## 5. Conclusion

Summarily, the pes anserinus as found in this study is at variance with earlier descriptions both morphologically and morphometrically. It was not layered. It was formed by semitendinosus, semimembranosus, gracilis, and sartorius tendons, their accessory tendon bands, and tibial collateral ligament. Meanwhile, the accessory tendon band may produce premature tendon graft. This morphometric marking described can help to limit donor site injury, nerve injuries, and postoperation hamstring weakness. We also found that pes anserinus possessed prolonged distal attachment which will be of improved mechanical advantages.

## Figures and Tables

**Table 1:** Classification (types) of pes anserinus.

Classification	Classes	Number (20)	Total (100%)
Based on tendinous formation	Type I (SAT + GRT + STT)	1	5%
	Type II (SAT + GRT + STT+ aSTT + SMT + TCL)	1	5%
	Type III (SAT + GRT + STT + aSTT)	13	65%
	Type IV (SAT + GRT + STT + aSTT + SMT)	3	15%
	Type V (SAT + STT + GRT + aSAT + SMT)	1	5%
	Type VI (SAT + GRT + STT + aGRT + aSTT)	1	5%

Based on union of the tendons	Nonconvergence (side-by-side termination)	2	10%
	Early convergence (superior to medial tibial condyle)	1	5%
	Late convergence (inferior to medial tibial condyle)	17	85%

Based on point of insertion	Superior anteromedial tibia	0	0%
	Medial border of tibia	0	0%
	Superior anteromedial bone + medial border of tibia	18	90%
	Superior anteromedial bone + medial border of tibia + fascia cruris	2	10%

(i) SAT: sartorius muscle tendon.

(ii) GRT: gracilis muscle tendon.

(iii) STT: semitendinosus muscle tendon.

(iv) SMT: semimembranosus muscle tendon.

(v) aSAT: accessory sartorius muscle tendon.

(vi) aSTT: accessory semitendinosus muscle tendon.

(vii) aGRT: accessory gracilis muscle tendon.

**Table 2:** Mean, minimum, and maximum values of pes anserinus morphometric parameters.

S/N	Pes anserinus morphometric parameter	Mean value	Minimum value	Maximum value
1	Minimum horizontal distance (mm)	24.96	10.06	39.40

2	Maximum horizontal distance (mm)	65.03	48.99	88.92

3	Lowest insertion point relative to tibial tuberosity (mm)	124.44	24.71	222.77

4	Proximodistal insertion distance (mm)	134.02	20.30	299.69

5	Mediolateral distance relative to tibial tuberosity (mm)	40.36	22.76	71.78

**Table 3:** Mean values for the length and thickness of the sartorius, gracilis, and semitendinosus tendons between specimens.

Parameters	Total	Males	Females
Sartorius tendon			
Length (mm)			
Left	53.14	53.57	51.63
Right	52.18	50.02	59.74
Left + right	52.66	51.79	55.68
Thickness (mm)			
Left	1.55	1.55	1.03
Right	1.43	1.59	1.39
Left + right	1.49	1.57	1.21

Gracilis tendon			
Length (mm)			
Left	120.72	118.10	129.85
Right	111.43	109.14	119.43
Left + right	116.07	113.63	124.64
Thickness (mm)			
Left	1.97	1.90	2.23
Right	1.87	1.74	2.31
Left + right	1.92	1.82	2.27

Semitendinosus tendon			
Length (mm)			
Left	133.07	131.16	139.76
Right	130.04	127.57	138.73
Left + right	116.80	129.36	139.25
Thickness (mm)			
Left	2.92	2.94	2.84
Right	2.77	2.74	2.9
Left + right	2.66	2.84	2.87

There was no statistical difference between male and female tendon (sartorius, gracilis, and semitendinosus) lengths and thicknesses.
